# A Clinical Analysis of Thirty-Five Patients Undergoing Aortic Reoperation

**DOI:** 10.21470/1678-9741-2020-0287

**Published:** 2022

**Authors:** Xin Yuanfeng, Jian Kaitao, Safwa Mahmood, Liu Jianshi, Sun Lizhong, He Yaping, Liu Wei

**Affiliations:** 1 Department of Cardiovascular Surgery, Shanghai East Hospital, Tongji University, Shanghai, People’s Republic of China.; 2 Department of Cardiovascular Surgery, Shanghai Deltahealth Hospital, Shanghai, People’s Republic of China.; 3 Department of Cardiovascular Surgery, Shanghai 10th People’s Hospital, Tongji University, Shanghai, People’s Republic of China.; 4 Department of Cardiology, Shanghai Jiahui International Hospital, Shanghai, People’s Republic of China.

**Keywords:** Reoperation, Aneurysm, Aortic Dissection, Aorta, Vascular Surgical Procedures, Hospital Mortality

## Abstract

**Introduction:**

At present, there are few reports regarding the issue of aortic reoperation due to its complexity and high risk and individual differences among patients.

**Methods:**

From November 2016 to December 2017, the data from 35 cases of aortic reoperation at our institution, out of 212 consecutive aortic repairs, were reviewed. We retrospectively summarized and analyzed their surgical indications, operative data, time interval from previous aortic repair, and outcomes. The time intervals until reoperation were analyzed for differences.

**Results:**

Patients’ mean age was 40.9±14.5 years, and 25 of them were men (71.4%). The indications for reoperation were aortic valvular problem (14.3%), aneurysmal dilatation (25.7%), pseudoaneurysm formation due to anastomotic leakage (43.2%), and aortic dissection (17.1%). For patients who had underwent primarily emergency operations due to aortic dissection, the time interval until reoperation (4.8±3.2 years) was significantly shorter than that of the whole group (5.5±3.6 years, *P*<0.01). Among the 35 reoperations, Sun’s procedure was selected for 16 patients (45.7%) with total aortic arch reconstruction. The average follow-up was 12 months (range 9-15 months). Hospital mortality was 5.7% (two patients). Among the hospital survivors there were no cases of death, rupture of residual dissection, paraplegia, or central nervous system complications during the follow-up period.

**Conclusion:**

Patients with acute aortic dissection required repeat surgery significantly earlier compared to other diseases. As to reoperation strategy, we recommend Sun’s procedure as the choice for extended arch reconstruction since minimal effect on overall mortality and complication rates were found.

**Table:** 

Abbreviations, acronyms & symbols	
Avg	= Average
CABG	= Coronary artery bypass grafting
CNS	= Central nervous system
CT	= Computed tomography
IABP	= Intra-aortic balloon pump
ICU	= Intensive care unit
NYHA	= New York Heart Association
Scr	= Serum creatinine
SD	= Standard deviation

## INTRODUCTION

During the past 10 years, a better understanding of various aneurysms, continuous improvements of surgical strategies and aortic techniques, and the emergence of new technologies and instruments have diversified the treatment of aortic aneurysms in China. However, due to the complexity of aortic disease, which is often multiple and recurrent in nature, aortic reoperation or multiple operations are quite common. At present, awareness about reoperation in aortic surgery has also increased, and several retrospective studies on aortic reoperation after acute dissection have surfaced in the recent years. Moreover, indications for a second surgery can be summarized as: progressive aortic insufficiency ^[1]^,progressive expansion of retained aortic root ^[2]^, distal pseudoaneurysm dilatation ^[3,4]^, poor perfusion syndrome ^[5]^, anastomotic leakage at proximal and distal ends of prosthesis or coronary anastomotic leakage after Bentall operation ^[6]^, and infection and structural failure or endovascular stent.

Due to the complexity of aortic reoperation and individual differences among patients, there are currently few reports regarding this issue in the literature, and most of them are single-center reports with less than 30 cases ^[6]^. Advanced age, Marfan syndrome, and aortic dissection are the high-risk factors for aortic reoperation intervention. The rate of aortic reoperation intervention varies from 7% to 25%, and mortality rate varies from 5% to 20%. Recently, a report of a larger sample size came from Eduard Quintana, of Mayo Clinic, who reported that among 650 patients of aortic arch surgery, 26% (172) underwent aortic reoperation. A retrospective analysis of 168 of these 172 patients showed that their 30-day mortality rate was 8.3% and the incidence of thromboembolism was 5.4% ^[7]^. Also, a recent report in North America showed that mortality and embolization rates of aortic reoperation were 11.6% and 11%, respectively ^[8]^. However, there are currently very few reports about this kind of reoperation in China. Hence, we retrospectively summarized 35 cases of aortic reoperation and analyzed their causes, surgical methods, and results, attempting to accumulate data as well as experience for secondary aortic surgery.

## METHODS

### Study Subjects

This is a retrospective study. Thirty-five patients (16.5%) underwent reoperation in a total of 212 patients of aortic surgery in Shanghai Deltahealth Hospital between November 2016 and December 2017. Cases of infection and damage of vascular grafts or endovascular stents were excluded from the study. Reoperations mainly involved the ascending aorta and the aortic arch (median incision); reoperations for thoracic descending aorta and thoracoabdominal aortic aneurysm (lateral thoracic incision) were not included in this study. This study focused on unplanned reoperations which were closely correlated with the first operations. These reoperations are secondary to distal progression of aortic disease or complications of previous procedures. Therefore, we excluded scheduled reoperations for descending thoracic aorta or abdominal aorta, for example, in patients with Marfan syndrome. This study was approved by the Ethics Committee of Deltahealth Hospital Shanghai - approval number SDH (2018) KYLWPJ 001.

### Preoperative Evaluation and Operation Strategy

All 35 patients underwent preoperative aorta computed tomography (CT) angiography and echocardiography. The CT imaging was focused on the diameter of different parts of aorta, anatomical characteristics of each aortic branch, residual aortic dissection and atherosclerotic plaque, and the distance between the sternum and thoracic structures. Echocardiography was focused on valve regurgitation, shunt location and distance from proximal and distal anastomotic leakage, combined with specific localization by CT, which was critical for decision of surgical strategy.

In order to ensure the safety of the procedure, all the operations were performed under general anesthesia, routine central venous catheterization, nasopharyngeal and bladder temperature monitoring, and continuous cerebral oxygen saturation monitoring. A midline incision was performed during reoperation for all the patients in this group, femoral artery and vein or axillary artery were exposed. A preoperative CT evaluation of the distance between the sternum and vital organs was mandatory. If the distance between the sternum and vital organs was < 0.5 cm, thoracotomy was performed only after direct femoral arteriovenous cannulation. Moreover, for venous blood return, catheterization was performed in either femoral vein or right atrium. After aortic occlusion, anterograde myocardial perfusion was instituted followed by direct perfusion into left and right coronary artery orifices. For patients who required aortic arch reconstruction, deep hypothermic circulatory arrest with nasopharyngeal temperature at 22-25℃ was adopted. Anterograde unilateral cerebral perfusion (5 ml/kg/min) was administered to all patients, and bilateral cerebral oxygen changes were closely monitored while administering it. In patients with high-risk factors for circulation deficiency in Circle of Willis, intraoperative cerebral oxygenation was not satisfactory, and an immediate change to bilateral anterograde cerebral perfusion was started.

### Follow-up

Follow-up evaluation included aortic CT angiography prior to discharge, reevaluation of echocardiography, telephone follow-up, and online follow-up. Moreover, at six and 12 postoperative months there were another aortic CT angiography and echocardiography to check the valvular condition, cardiac function, aortic graft, and condition of thrombosis at distal residual dissection.

### Statistical Analysis

All values are expressed as the mean ± standard deviation or percentages. Differences between patient groups were tested by univariate analysis (two-tailed *t*-test). Findings of *P*<0.05 were considered statistically significant; *P*<0.01 was considered very statistically significant. All analyses were performed using the IBM Corp. Released 2015, IBM SPSS Statistics for Windows, Version 23.0, Armonk, NY: IBM Corp. software.

## RESULTS

For detailed clinical data please refer to Table 1. The reoperation cohort consists of 25 males (71.4%) with an average age of 40.9±14.5 years. Among them, two (5.7%) underwent emergency operation and 18 (51.4%) had Marfan syndrome; six patients (17.1%) underwent a third aortic operation. First operations were all performed in other hospitals, the methods and indications of those operations were summarized in Table 2. The main causes of reoperation were: 1) aortic valve-related factors (including valve regurgitation, structural and perivalvular leakage) in five (14.3%) patients; 2) aneurysmal dilatation of aortic root in four (11.4%) patients; 3) pseudoaneurysm formation in proximal anastomotic leakage (including coronary anastomotic leakage) in 11 (31.8%) patients; 4) aneurysmal dilatation of aortic arch in five (14.3%) patients; 5) pseudoaneurysm formation in distal anastomotic leakage in four (11.4%) patients; and 6) redissection in six (17.1%) patients (Table 3).

**Table 1:** Baseline clinical characteristics of 35 cases of reoperation.

Basic information	n	%
Male	25	71.4
Age (years) (avg±SD)	40.9±14.5	NA
NYHA Class III-IV	5	14.3
Chronic lung disease	1	2.8
Scr > 2.0 mg/dl	0	0
Stroke	1	2.8
Hypertension	15	42.8
Diabetes	5	14.3
Coronary artery atherosclerosis	2	5.7
Marfan syndrome	18	51.4
Behcet's disease	1	2.8
Emergency operation	2	5.7

Avg=average; NYHA=New York Heart Association; Scr=serum creatinine; SD=standard deviation

**Table 2:** Indications and methods of the first aortic operation.

	n	%
**Indications for the first operation**		
Acute type A aortic dissection	17	48.6
Aortic aneurysm	14	40
Infective endocarditis	2	5.7
Leakage after stent implantation	1	2.8
Aortic regurgitation	1	2.8
**Methods of the first operation**		
Ascending aorta replacement + Sun's procedure	8	22.8
Ascending aorta + aortic valve replacement	1	2.8
Ascending aorta replacement	1	2.8
Ascending aorta + half-arch replacement + CABG	1	2.8
Bentall procedure	13	37.1
Bentall + Sun's procedure	3	8.6
Bentall + mitral valve replacement	1	2.8
Bentall + trifurcated graft placement	1	2.8
Bentall + CABG	2	5.7
David procedure	1	2.8
Aortic valve replacement	3	8.6

CABG=coronary artery bypass grafting

**Table 3:** Indications for the reoperation.

Indications for the reoperation	n	%
Aortic valve-related factors	5	14.3
Aneurysmal dilatation of aortic root	4	11.4
Pseudoaneurysm formation in proximal anastomotic leakage	11	31.8
Aneurysmal dilatation of aortic arch	5	14.3
Pseudoaneurysm formation in distal anastomotic leakage	4	11.4
Redissection	6	17.1

In this study the average interval of reoperation was 5.5±3.6 years. The time interval between first emergency operation for acute type A aortic dissection and reoperation was 4.8±3.2 years, which was significantly shorter than that of the entire group (P<0.01, Table 4).

**Table 4:** Time interval between the first surgery and reoperation.

	All cases	Acute type A aortic dissection as first operation
Time interval (years)	5.5±3.6	4.8±3.2

*P*-value refers to the comparison between the two groups.

Details of the methods of reoperation are listed in Table 5. There were nine cases (25.7%) of direct repair of anastomotic leakage, and 16 cases (45.7%) were treated with variants of the Sun’s procedure. The Sun’s procedure is a surgical technique proposed by Dr. Li-Zhong Sun in 2002 that integrates total aortic arch replacement using a tetrafurcated graft with implantation of a specially designed frozen elephant trunk (Cronus®) in the descending aorta. It is used as a treatment option for extensive aortic dissections or aneurysms involving the ascending aorta, aortic arch, and the descending aorta. Sun’s procedure plays an important role in aortic reoperation, especially in reoperations of the aortic arch ^[9,10]^. In 10 cases (28.6%), direct femoral arteriovenous cannulation was first performed, and three cases (8.6%) had rupture of the original vascular prosthesis during thoracotomy. When rupture of vital organs occurred during thoracic entry, the sternum was closed quickly with towel clips, and femoral arteriovenous catheterization was carried out immediately. Axillary artery was used in two cases (5.9%), femoral artery in 26 cases (76.5%), and ascending aorta in six cases (17.6%).

**Table 5:** Methods of reoperation.

Method of reoperation	n	%
**Repair of anastomotic leakage**		
Proximal anastomotic leakage (includes coronary artery anastomotic leakage)	6	17.1
Distal anastomotic leakage	2	5.7
Periaortic valve leakage	1	2.8
Sun's procedure	2	5.7
+ ascending aorta replacement	10	28.6
+ aortic valve replacement	1	2.8
+ mitral valve replacement	1	2.8
+ abdominal aorta replacement	1	2.8
+ Carbrol procedure	1	2.8
Bentall procedure	3	8.6
Ascending aorta replacement	2	5.7
Aortic valve replacement	1	2.8
**Excision of pseudoaneurysm**		
+ aortic valve replacement	1	2.8
+ ascending aortic perigraft-to-right atrial shunt	1	2.8
+ tricuspid valvuloplasty	1	2.8
Apico-brachiocephalic artery bypass + aortic stent implantation	1	2.8
CABG + resection of ventricular aneurysm	1	2.8

CABG=coronary artery bypass grafting

From the entire group of patients, 34 (97.1%) underwent cardiopulmonary bypass during reoperation, the mean cardiopulmonary bypass time was 157.9±58.8 minutes, and aortic occlusion time was 91.3±31.8 minutes. Of these 34 cases, deep hypothermic circulatory arrest was adopted in 18 cases (51.4%) and the mean time of cerebral perfusion after circulatory arrest was 21.7±9.3 minutes. Postoperative intensive care unit (ICU) stay for over 10 days occurred in two cases (5.7%). The remaining 33 patients (94.3%) had an average of 2.3±1.1 days of ICU stay. Time on ventilator was 6.4±4.2 hours, including one case of reintubation (2.85%), one case of rethoracotomy (2.85%, coronary artery bypass grafting [CABG] + resection of ventricular aneurysm), and one case of intra-aortic balloon pump (IABP) implantation (2.85%). No case of rethoracotomy for hemostasis due to excessive drainage was reported. There was no case of permanent pacemaker implantation. Mean intraoperative erythrocyte transfusion was 3.5±2.7 units.

Of the 35 patients reoperated, two patients (5.7%) died in the hospital and stroke occurred in two patients (5.7%) within 30 days postoperatively. The first mortality case was a patient who underwent surgery eight years before for aortic root aneurysm and came for reoperation due to formation of a giant pseudoaneurysm (53 mm × 75 mm) on the ascending aorta. The main left coronary artery involved was unclear on CT, and pulmonary artery and left atrium were obviously compressed. Intraoperative exploration revealed absence of left coronary artery opening in the left coronary sinus, and a giant pseudoaneurysm could be seen under the right pulmonary artery. It was found that the body of the aneurysm was connected to the coronary artery being incised. An excision of the pseudoaneurysm and a tricuspid valvuloplasty were performed. However, there was a post-pericardiotomy low cardiac output (echocardiographic indication due to pulmonary artery compression) and the patient died on the fifth day after operation. The other patient underwent Bentall operation three years before. Reoperation was performed for aneurysmal dilatation of ascending aorta (diameter 62 mm) and anastomotic leakage of left coronary artery (3 mm). The patient underwent repair of coronary artery leakage, but low cardiac output appeared on the first day after operation. An IABP was implanted, but still a left ventricular aneurysm appeared on echocardiography. Considering the unsatisfactory results of the coronary artery repair, CABG + ventricular aneurysm resection were performed again on the 17^th^ day after operation and the patient died on the 20^th^ day. Postoperative brain complications occurred in two cases (5.7%), one with delirium and other brain symptoms, and the other with multiple small-area cerebral infarctions; both patients recovered smoothly after treatment and were discharged from hospital.

All hospital survivors were discharged smoothly and were followed up for an average of 12 months (range 9-15 months). There were no cases of death, rupture of residual dissection, paraplegia, or central nervous system complications during the follow-up period up to 15 months postoperatively (Table 6).

**Table 6:** Post-reoperative outcomes.

	30 days (n)	12 months (n)
Mortality	2	0
CNS disorder	2	0
Paraplegia	0	0
Rupture of residual dissection	0	0

CNS=central nervous system

## DISCUSSION

Reoperation of the aorta is an issue of great complexity and variability, this is correlated with the diversified techniques used in the first operation, each of which presents with different clinical and anatomical characteristics. This is especially true when the first operation was an emergency aortic dissection surgery. Due to the diverse pathologic features in different aortic segments, the selection of surgical technique would be highly individualized. However, in general, the main goals of the first operation are: 1) avoiding rupture of aortic aneurysm; 2) curing pericardial tamponade; 3) resuming adequate and effective blood perfusion to minimize malperfusion. Through 20 years of follow-up, Piccardo A et al. ^[11]^ verified that simple ascending aortic replacement (above the coronary orifice) or additional aortic valve suspension technique have showed good reliability. In recent years, with the continuous progress of brain protection technology ^[12-14]^, more medical centers began to ignore the primary tear site and directly perform a full arch elephant trunk procedure. Some researchers believe that a more thorough surgical procedure during the first operation will reduce the rate of reoperation in the proximal and distal ends, as well as improve long-term survival ^[4,15-17]^. On the other hand, it has not been shown to decrease the probability of reoperations significantly and was even associated with increased risk in a few clinical researches ^[16,18]^. Obviously, the persistent issue is still of debate. In fact, routine extension of replacement into the total aortic arch in the initial operation has not been adopted in most medical centers currently ^[19]^. It is more advisable for connective tissue disease. In our medical center, we tend toward a more aggressive approach using total replacement of the ascending aorta and aortic arch combined with stented elephant trunk implantation (Sun’s procedure) in patients with acute type A dissection. One important consideration is that in China patients with acute type A dissection are of younger age than those in western countries. The mean age of Chinese patients is in the forties ^[20]^. The common causes of aortic dissection are uncontrolled hypertension instead of degenerative aneurysm. Young patients and less degenerative aorta may contribute to lower perioperative mortality with the more invasive procedure. This supposition lacks evidences and needs substantial clinical data in the future.

There are still very few reports about aortic reoperation in China and worldwide; moreover, reoperation patients recruited in existent researches were of highly varied aortic pathology, and most researches had limited sample sizes. Schäfers et al. ^[21]^ reported that 10 years after the first operations on acute type A aortic dissection, the probability of avoiding reoperation was about 70%-80%, the mortality of proximal end aorta reoperation was about 10%, and the mortality of distal end aorta reoperation was about 0-4%. Kirsch et al. ^[22]^ summarized 160 cases of surgical treatment of type I aortic dissection; among them, 30 patients (18.8%) underwent 36 reoperations. Recently, statistics from a large cohort of reports showed that the rate of reoperation after acute dissection was 20.2% (47/232), the average interval time between operations was 5.2±5.3 years, and the mortality rate was 7.7% ^[23,24]^. Moreover, the main cause of reoperation of aorta includes (disregarding planned phase 2 thoracoabdominal aortic surgery): valve regurgitation, dilated aneurysms proximal or distal to the surgical site on the arteries, proximal and distal anastomotic leakage (coronary anastomotic leakage), pseudoaneurysm formation, a new aortic dissection or aneurysm, and infection of vascular prosthesis, etc.

In our current study, the group of 35 patients who underwent reoperation accounted for 16.5% of all major vascular operations in our center. Because all the 35 patients underwent the first operation in other medical services and were referred to our hospital, we could not infer the reintervention rate. However, during the outpatient clinic and follow-up visits, we discovered that a considerable number of patients chose to abandon surgery and switch to conservative treatment despite the need for reoperation, this is either due to their fear of uncertainty towards the reoperation or for financial reasons. Moreover, this part of the data is not included in the statistics, thus the reoperation rate of major vascular surgery in China needs further investigation based on multicenter studies in the future. In this study, the average interval of reoperation was 5.5 ±3.6 years. The time interval between first operation for emergency dissection and reoperation was 4.8±3.2 years, which was significantly shorter than that of the entire group. Moon ^[16]^ and Bachet ^[25]^ also found that primary emergency operation for acute type A dissection was a risk factor for reoperation and these surviving patients tended to require repeat operations much earlier compared with those who did not undergo primary emergency operations. Therefore, if the first operation is an emergency dissection, we should pay more attention to the choice of surgical method and the perfection of surgical technique. According to several clinical researches in China, Sun’s procedure showed to be an effective treatment option for extensive and complex aortic repairs ^[26-28]^. It is reasonable to suppose that the application of Sun’s procedure might reduce the reoperation rate in the long term.

Thorough understanding of the first operation is an important factor for choosing the appropriate strategy for aortic reoperation, as well as in the consideration of method and extent of management ^[12,29,30]^. For a second thoracotomy, it is very important to perform a preoperative CT to evaluate the posterior sternal distance, especially to evaluate the gap between the sternum and vascular prosthesis. If the distance is < 0.5 cm, we recommend performing thoracotomy only after direct femoral arteriovenous cannulations. Furthermore, in case of rupture of vital organs or vascular prosthesis, the sternum can be closed quickly with towel forceps, and emergency femoral arteriovenous cannulation and catheterization should be carried out simultaneously. Also, the use of a balloon catheter in this group has also achieved good temporary hemostasis in the absence of massive bleeding.

No matter which kind of operation method was selected, each patient in this study was prepared for deep hypothermic circulatory arrest, routine cerebral oxygen monitoring, and transesophageal echocardiography. It would be easier to identify intraoperatively the problem of the first operation. Especially for patients with anastomotic leakage complicated with pseudoaneurysm formation, location of the leakage by transesophageal echocardiography was subsequently verified by intrathoracic and extracardiac exploration during surgery. Transesophageal echocardiography played a very important role in guiding the specific operation in the following ways: enhancing the certainty and reliability of the repair of anastomotic leakage, avoiding a series of side injuries caused by repeated operation, and avoiding blindly enlarging the extent of the operation, thus reducing the complexity and difficulty of the operation for better outcome.

Owing to the selection of appropriate surgical methods and continuous comprehensive surveillance, this group of patients achieved good clinical results. In this group, the mortality rate was 5.7% and the incidence of complications was also 5.7%. Cardiopulmonary bypass time was 157.9±58.8 minutes and aortic occlusion time was 91.3±31.8 minutes. The length of cerebral perfusion after circulatory arrest was 21.7±9.3 minutes, ventilator-assisted time was 6.4±4.2 hours, and duration of ICU stay was 2.3±1.1 days. Compared with similar reports in the literature, this is a relatively good experience ^[7,27]^. In this group, 16 cases (45.7%) underwent reoperation using Sun's procedure. We believe that the overall mortality and complication rates were not affected by extended arch reconstruction during aortic reoperation. Reoperations to address the aortic arch have acceptable short-term mortality and morbidity based on our data. In addition, with regards to the repair of coronary anastomotic leakage or proximal and distal leakage, transesophageal echocardiography monitor reduced the difficulty of the operation, avoided cardiopulmonary bypass, simplified the technique, ensured the effectiveness of operation, and reduced the side effects of reoperation. The two cases of death in this group were reoperations involving the coronary artery, so more caution should be emphasized to the reoperations involving the coronary artery. In cases with concomitant coronary complications, may aggressive coronary bypass be considered to reduce mortality and morbidity rates? We will closely observe and follow-up these cases in our future work.

### Limitations

There are some limitations in this report, including the short follow-up time, the diversification of surgical methods used in the first operation, and the different reasons for reoperation, all these will certainly have an impact on our results. In future studies, we will strive to further expand the sample size, to extend the follow-up time, and to look for new key findings in different methods of aortic reoperation.

## CONCLUSION

Patients who underwent operation for acute aortic dissection required repeat surgery significantly earlier compared to other diseases. As to reoperation strategy, we recommend the procedure of choice for extended arch reconstruction to be Sun's procedure, since minimal effect on overall mortality and complication rates were found.

**Table:** 

Authors' roles & responsibilities	
XY	Substantial contributions to the conception and design of the work; and analysis and interpretation of data for the work; final approval of the version to be published
JK	Substantial contributions to the analysis of data for the work; drafting the work or revising it critically for important intellectual content; final approval of the version to be published
SM	Drafting the work or revising it critically for important intellectual content; final approval of the version to be published
LJ	Final approval of the version to be published
SL	Final approval of the version to be published
HY	Substantial contributions to the analysis of data for the work; final approval of the version to be published
LW	Final approval of the version to be published
